# Enhanced Avoidance Habits in Relation to History of Early-Life Stress

**DOI:** 10.3389/fpsyg.2019.01876

**Published:** 2019-08-13

**Authors:** Tara K. Patterson, Michelle G. Craske, Barbara J. Knowlton

**Affiliations:** Department of Psychology, University of California, Los Angeles, Los Angeles, CA, United States

**Keywords:** stress, habit, avoidance learning, instrumental learning, outcome devaluation, goal-directed action

## Abstract

The effect of stress on the balance between goal-directed behavior and stimulus–response habits has been demonstrated in a number of studies, but the extent to which stressful events that occur during development affect the balance between these systems later in life is less clear. Here, we examined whether individuals with a history of early-life stress (ELS) show a bias toward avoidance habits on an instrumental learning task as adults. Participants (*N* = 189 in Experiment 1 and *N* = 112 in Experiment 2) were undergraduate students at the University of California, Los Angeles. In Experiment 1, we hypothesized that a history of ELS and a longer training phase would be associated with greater avoidance habits. Participants learned to make button-press responses to visual stimuli in order to avoid aversive auditory outcomes. Following a training phase involving extensive practice of the responses, participants were tested for habitual responding using outcome devaluation. After completing the instrumental learning task, participants provided retrospective reports of stressful events they experienced during their first 16 years of life. We did not observe evidence for an effect of the length of training, but we did observe an effect of ELS, with greater stress predicting greater odds of performing the avoidance habit. In Experiment 2, we sought to replicate the effect of ELS observed in Experiment 1, and we also tested whether the presence of distraction during training would increase avoidance habit performance. We replicated the effect of ELS but we did not observe evidence of an effect of distraction. Taken together, these data lend support to the hypothesis that stress occurring during development can have lasting effects on the balance between goal-directed behavior and stimulus–response habits in humans. Enhancement of avoidance habits may help explain the higher levels of negative health outcomes such as heart and liver disease that have been observed in individuals with a history of ELS. Some of the negative health behaviors that contribute to these negative health outcomes, e.g., overeating and substance use, may be performed initially to avoid feelings of distress and then transition to being performed habitually.

## Introduction

The effects of stress on physical and psychological health have been of increasing interest in recent years, with one area of focus being how individuals are affected by stress that occurs during development (early-life stress, ELS). Common sources of ELS are childhood abuse and neglect. Such experiences have been shown to cast a long shadow on health throughout the lifespan, affecting outcomes in adulthood ranging from severe obesity (Anda et al., 2006), heart disease (Dong et al., 2004), and liver disease (Dong et al., 2003) to sexually transmitted disease (Hillis et al., 2000) and depressive disorders (Chapman et al., 2004). The behavioral and neural mechanisms of the associations between ELS and adult health are largely unknown. Because many negative health outcomes are linked to repetitive behaviors such as overeating or substance use, it is possible that an increased reliance on stimulus–response habits in this population could explain some of the health effects experienced by its constituents.

Stimulus–response habits can be defined as instrumental behaviors that, in contrast to goal-directed actions, have come to be automatically elicited by stimuli in whose presence the behavior has been repeatedly performed, without regard to instrumental outcomes (Dickinson, 1985). For example, an animal that has been overtrained to press a lever to obtain a food reward will persist in lever pressing even after the food outcome has been devalued (Adams, 1982). In this scenario, the animal’s behavior is thought to be guided by the stimulus–response association (i.e., the association between the lever and the pressing behavior) rather than by the value of the outcome (i.e., the food reward), because the animal persists in performing the response when the stimulus is present even though the outcome associated with performing that response is no longer desired. Habits have also frequently been studied using maze navigation tasks, especially in rodents; in this assay, habitual behavior is assessed by setting up a situation where the extent to which behavior is based on stimulus–response associations can be inferred from navigation decisions or performance (e.g., Packard and McGaugh, 1992; McDonald and White, 1994). In humans, habitual behavior has also been investigated with the probabilistic classification task, in which participants learn to classify stimuli based on trial-by-trial feedback. This task can be performed using the habit memory system, as in the case of individuals with amnesia (Knowlton et al., 1994), and can also be performed using the declarative memory system, as in the case of individuals with Parkinson’s disease (Knowlton et al., 1996).

A number of studies have shown that stress increases habitual behavior in both non-human animals and humans. Experimentally induced stress has been shown to decrease sensitivity to outcome devaluation (Dias-Ferreira et al., 2009; Schwabe and Wolf, 2009, 2010), increase habitual behavior in maze navigation (Kim et al., 2001; Schwabe et al., 2008), and bias competition between the declarative memory system and the habit learning system in favor of habit learning in probabilistic classification (Schwabe and Wolf, 2012). The effects of stress on habitual behavior are likely mediated by stress hormones (for review, see Wirz et al., 2018), and a study using human infants showed that this stress-induced shift to habitual responding can occur as early as 15 months of age (Seehagen et al., 2015). Although most studies of this phenomenon have measured habitual behavior shortly after stress exposure, a study of male rats exposed to stress during the first 2 weeks of life found that they showed increased habitual behavior in maze navigation as adolescents (Grissom et al., 2012), and humans whose mothers reported that they were exposed to stress prenatally showed increased habitual behavior in maze navigation as adults (Schwabe et al., 2012).

Two other factors that have been shown to influence habitual behavior are the amount of training and the presence of distraction. Animals that receive a limited amount of training show behavior that is goal-directed (i.e., sensitive to outcome devaluation), whereas animals that receive extended training show behavior that is habitual (i.e., insensitive to outcome devaluation), indicating that with greater training, behavior transitions from being controlled by action–outcome associations to being controlled by stimulus–response associations (Adams, 1982; Dickinson, 1985). One study has successfully demonstrated this effect in humans, showing that participants who received limited training were sensitive to outcome devaluation whereas participants who received extended training were not (Tricomi et al., 2009). A second factor that appears to influence habits is distraction. For example, in the probabilistic classification task, the presence of distraction by a secondary task appears to bias competition between the declarative memory system and the habit learning system in favor of habit learning (Foerde et al., 2006, 2007).

Stimulus–response habits can be appetitive (e.g., pressing a lever to receive a food reward) or avoidant (e.g., pressing a lever to avoid a shock). Most research on stimulus–response habits has been conducted using appetitive habits, as the methods for evaluation of habit formation through devaluation of appetitive outcomes via conditioned taste aversion or selective satiation procedures have been well established (for review, see Knowlton and Patterson, 2018). However, in a pair of studies conducted by Gillan et al. (2014, 2015), a shock avoidance task incorporating a novel procedure for devaluation of aversive outcomes was used to investigate avoidance habits. In this task, participants learned to avoid electric shocks delivered to the left and right wrist by making responses to warning stimuli with the left and right foot, respectively. Next, one of the two outcomes was devalued by disconnecting one of the electrodes and leaving the other electrode connected. Participants’ responding to the valued and devalued stimuli was then tested in extinction. Selective responding to the still-valued stimulus indicates that participants have flexibly adjusted their behavior (i.e., that they are behaving in a goal-directed manner), whereas persistence in responding to the devalued stimulus despite the built-in cost to performance that results from continuing to hold in mind a rule that no longer applies and executing unnecessary behaviors on the basis of this rule is interpreted as habitual behavior. Using this procedure, Gillan et al. (2014, 2015) demonstrated enhanced avoidance habits in individuals with obsessive-compulsive disorder. Like compulsions, some negative health behaviors such as overeating and substance use can be understood as avoidance habits, because they may be performed initially in order to avoid feelings of distress, and then eventually transition to being performed habitually. We were therefore interested in whether adults with a history of ELS might also show enhanced avoidance habits. If so, this tendency could represent a behavioral vulnerability that increases the likelihood of the poor health outcomes observed in this group.

We used a noise avoidance task similar to the shock avoidance task used by Gillan et al. (2014, 2015), wherein participants could avoid hearing aversive noises delivered to the left and right ears by making the correct keyboard responses to associated warning stimuli. After learning the responses, participants underwent an instructed devaluation procedure in which one of the two earphones previously delivering aversive noises was removed, and then a test for habit formation was conducted in extinction. Avoidance habit formation was measured by whether the participant persisted in making the keyboard response associated with avoiding noise to the ear from which the earphone had been removed. In addition to testing for an effect of ELS, we also manipulated the level of training participants received (Experiment 1) and the level of distraction present during training (Experiment 2). The primary hypothesis of this study was that individuals who reported a history of ELS would show enhanced avoidance habits. The secondary hypotheses were (a) that individuals who received a greater level of training prior to devaluation would show enhanced avoidance habits relative to those who received less training, and (b) that learning the stimulus–response associations in the presence of distraction would lead to enhanced avoidance habits relative to associations learned without distraction.

## Experiment 1

### Materials and Methods

#### Participants

Study participants were recruited from the undergraduate student population in the Psychology Department at the University of California, Los Angeles. Participants were compensated with credit toward partial fulfillment of course requirements. Study procedures were approved by the Institutional Review Board of the University of California, Los Angeles, and all participants provided written record of informed consent.

A total of 198 participants were recruited for the study. Five participants did not complete the experiment, one participant failed to follow the instructions, two participants provided incomplete questionnaire data, and one data file was overwritten due to experimenter error, yielding a sample size of 189 (148 women, 41 men, *M*_age_ = 20.31 years, *SD*_age_ = 1.81 years, age range: 18–28 years).

#### Design and Procedure

**FIGURE 1:**
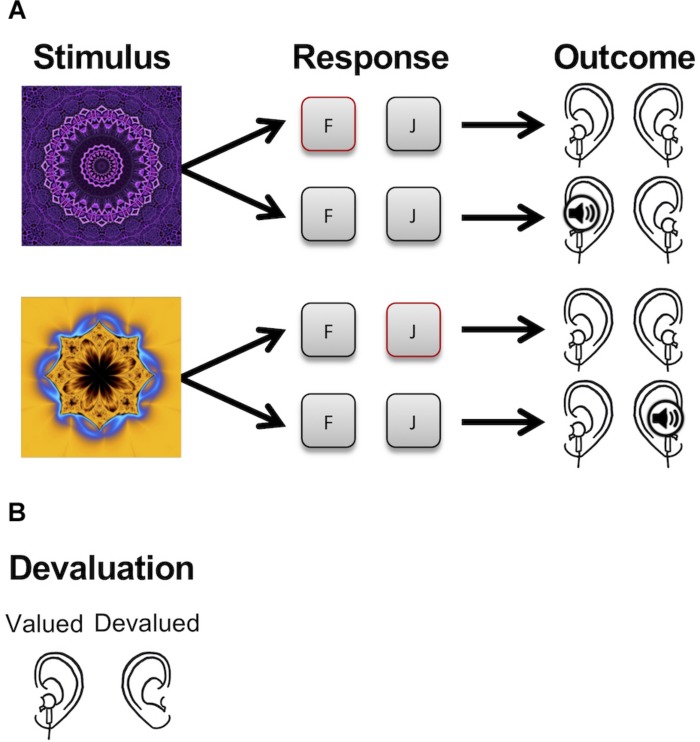
Task schematic. **(A)** Participants learned to make avoidance responses to two warning stimuli that predicted aversive noise played to the left (top) and right (bottom) earphones. If the correct avoidance response (shown in red) was made in time, the aversive noises were not delivered. **(B)** After training, one of the two outcomes was devalued by having participants remove one of the two earphones.

The avoidance learning task was adapted from procedures described in Gillan et al. (2014, 2015). A schematic of the task is shown in Figure 1. Participants were instructed that their task was to avoid hearing aversive noises. Participants were shown two abstract visual warning stimuli that predicted aversive noise to the left and right earphones, respectively, and were told that they could avoid hearing the aversive noises by making the correct keyboard responses when they saw the warning stimuli. Performing the correct response with the left hand avoided noise to the left earphone, and performing the correct response with the right hand avoided noise to the right earphone. A third stimulus was designated as the “safe” stimulus and never predicted aversive noise. Assignment of the three images to the three experimental trial types (warning stimulus 1, warning stimulus 2, and safe stimulus) was randomized across participants. On each trial, one of the three stimuli was selected randomly and presented on screen for 500 ms. Correct responses to the warning stimuli prevented aversive noise from being delivered to the earphones, but did not terminate the stimulus. If the participant pressed the incorrect key or failed to respond within 500 ms, the aversive noise (an audio file resembling a female scream) was delivered to the corresponding earphone. A female scream was selected as the aversive outcome based on the ease of implementation in comparison to an electric shock and based on prior research that used a female scream as an effective unconditional stimulus (e.g., Lau et al., 2008; Britton et al., 2011). Responses to the safe stimulus had no effect. There was a delay of 500 ms between termination of the warning stimulus and delivery of the aversive noise, and the intertrial interval was 2 s. Audio files were 1 s long and played at a volume of 82 dB.

Following demonstration of the stimulus–outcome contingencies, participants performed six practice trials (two per stimulus). Participants were allowed to repeat the practice phase if desired. The main experiment consisted of two phases, a training phase and a post-devaluation habit test. The amount of training was varied between subjects; participants in the short training condition completed 120 trials (40 per stimulus), and participants in the long training condition completed 600 trials (200 per stimulus). Assignment to condition was randomized across participants. After training was complete, one of the two outcomes was devalued by having participants remove one of the earphones. Which earphone was removed (left versus right) was counterbalanced across participants. Participants were told that they would be evaluated based on the responses they made to avoid noise to the earphone that had not been removed, and that it was not necessary to make the response associated with avoiding noise to the earphone that had been removed. The habit test was conducted in extinction (i.e., no noises were delivered to either earphone), but participants were not informed of this. The habit test consisted of 30 trials (10 per stimulus in random order). The dependent variable of interest was whether the participant persisted in performing the response associated with avoiding aversive noise to the removed earphone, as performance of this behavior was no longer of value and thus would be evidence of habit formation. Therefore, during the post-devaluation habit test, responding to the valued stimulus was defined as performing the response associated with avoiding aversive noise to the non-removed earphone when presented with the stimulus that had predicted aversive noise to the non-removed earphone, and responding to the devalued stimulus was defined as performing the response associated with avoiding aversive noise to the removed earphone when presented with the stimulus that had predicted aversive noise to the removed earphone.

Participants completed the experiment in a private testing room on a desktop computer. Stimulus presentation and response collection were implemented in E-Prime Standard (Version 2.0). Button press responses were made using the computer keyboard. Following completion of the computer task, participants completed a packet of questionnaires. The 25-item Childhood Trauma Questionnaire – Short Form (CTQ-SF; Bernstein et al., 2003) was used to assess stress exposure during the first 16 years of life. The items on the questionnaire ask about experiences of physical abuse (e.g., being hit hard enough to leave bruises), physical neglect (e.g., not having enough to eat), emotional abuse (e.g., being called names), emotional neglect (e.g., not feeling loved), and sexual abuse (e.g., being touched in a sexual way). Each item is rated on a 5-point scale with response options ranging from “never true” to “very often true.” The mean score reported by Bernstein et al. (2003) for this measure based on a normative community sample (*N* = 579) was 39.6. The 40-item State–Trait Anxiety Inventory (STAI; Spielberger, 1983) was used to assess anxiety at the present moment (state anxiety) and in general (trait anxiety). The 20-item Beck Depression Inventory-II (BDI-II; Beck et al., 1996) was used to assess depressive symptoms during the past 2 weeks (suicidality question omitted). Finally, the 10-item Perceived Stress Scale (PSS; Cohen et al., 1983) was used to assess how unpredictable, uncontrollable, and overloading participants’ lives had been during the past month. The entire lab visit took approximately 1 h.

#### Data Analysis

Statistical analyses were performed using IBM SPSS Statistics (Version 25). Data from the acquisition phase (response accuracy to the two warning stimuli and false alarm rate to the safe stimulus) and level of responding to the valued stimulus during the habit test were analyzed using two (level of training: 120 trials, 600 trials) × two (level of ELS: low-ELS, high-ELS) between-subjects ANOVA with participants categorized as low-ELS or high-ELS based on a median split of the CTQ-SF scores. Responding to the devalued stimulus during the post-devaluation habit test was analyzed using binary logistic regression with participants’ responses binned into zero responses to the devalued stimulus (no habitual behavior) or one or more responses to the devalued stimulus (habitual behavior). Responding was binarized in this manner based on a bimodal distribution of the response data among participants who responded to the devalued stimulus, with one subgroup of participants making few responses to the devalued stimulus and a second subgroup of participants responding on the majority of devalued stimulus trials. We therefore collapsed across the two subgroups, classifying all participants who responded to the devalued stimulus as exhibiting habitual behavior. The following predictors were included in the regression model: CTQ-SF, level of training, devalued side, STAI state anxiety, STAI trait anxiety, BDI-II, PSS, CTQ-SF × level of training, CTQ-SF × devalued side, CTQ-SF × STAI state anxiety, CTQ-SF × STAI trait anxiety, CTQ-SF × BDI-II, CTQ-SF × PSS, age, and gender. Continuous predictors used to create interaction terms were mean-centered to reduce multicollinearity, and dichotomous predictor variables were dummy coded. A significance level of 0.05 was used for all statistical tests.

A supplemental data analysis in which the number of responses to the devalued stimulus was entered as the outcome variable is provided in Supplementary Table 1.

### Results

Sample characteristics are reported in Table 1 (prevalence of ELS by degree and type of stress reported) and Table 2 (scores on questionnaire variables for low-ELS and high-ELS participants). The mean CTQ-SF score was 36.02 (*SD* = 11.98), and the median CTQ-SF score was 33.00. The low-ELS group had a mean CTQ-SF score of 27.96 (*SD* = 2.17) and the high-ELS group had a mean CTQ-SF score of 43.99 (*SD* = 12.38). The high-ELS group differed significantly from the low-ELS group on measures of state anxiety, *t*(187) = 5.37, *p* < 0.001, *d* = 0.78; trait anxiety, *t*(187) = 6.57, *p* < 0.001, *d* = 0.96; depression, *t*(187) = 6.96, *p* < 0.001, *d* = 1.01; and perceived stress, *t*(187) = 3.72, *p* < 0.001, *d* = 0.54.

**TABLE 1:** Prevalence of ELS in sample.

	**Experiment 1**					**Experiment 2**				
	**5**	**6–10**	**11–15**	**16–20**	**21–25**	**5**	**6–10**	**11–15**	**16–20**	**21–25**
**CTQ-SF subscale**										
Physical abuse	62.43	31.22	4.76	0.53	1.06	59.82	33.93	3.57	2.68	0.00
Physical neglect	46.56	43.92	8.47	1.06	0.00	53.57	46.43	0.00	0.00	0.00
Emotional abuse	31.22	47.62	14.81	2.65	3.70	25.89	57.14	10.71	6.25	0.00
Emotional neglect	26.98	47.09	17.46	6.88	1.59	29.46	47.32	17.86	4.46	0.89
Sexual abuse	84.66	11.11	1.59	1.59	1.06	84.82	9.82	2.68	0.89	1.79

*Percentage of participants in each experiment reporting Early-Life Stress (ELS) broken down by degree and type of ELS reported. For each subscale, five is the lowest score, corresponding to a response of “never” for all items. CTQ-SF, Childhood Trauma Questionnaire – Short Form (Bernstein et al., 2003).*

**TABLE 2:** Characteristics of low-ELS and high-ELS groups.

	**Experiment 1**		**Experiment 2**	
	**Low-ELS**	**High-ELS**	**Low-ELS**	**High-ELS**
**CTQ-SF**	27.96 (2.17)	43.99 (12.38)	27.80 (1.72)	41.15 (8.70)
**STAI**				
State anxiety	36.01 (10.76)	44.77 (11.62)	34.51 (12.68)	42.36 (12.64)
Trait anxiety	38.34 (9.75)	48.68 (11.78)	39.96 (11.13)	47.95 (11.57)
**BDI-II**	7.56 (6.11)	16.42 (10.73)	7.09 (7.09)	12.57 (9.33)
**PSS**	17.07 (6.32)	20.53 (6.51)	16.16 (6.44)	20.08 (7.31)

*Mean (*SD*) scores on questionnaire measures for participants grouped by reported level of childhood stress exposure. ELS, Early-Life Stress; CTQ-SF, Childhood Trauma Questionnaire – Short Form (Bernstein et al., 2003); STAI, State–Trait Anxiety Inventory (Spielberger, 1983); BDI-II, Beck Depression Inventory-II (Beck et al., 1996); PSS, Perceived Stress Scale (Cohen et al., 1983).*

**FIGURE 2:**
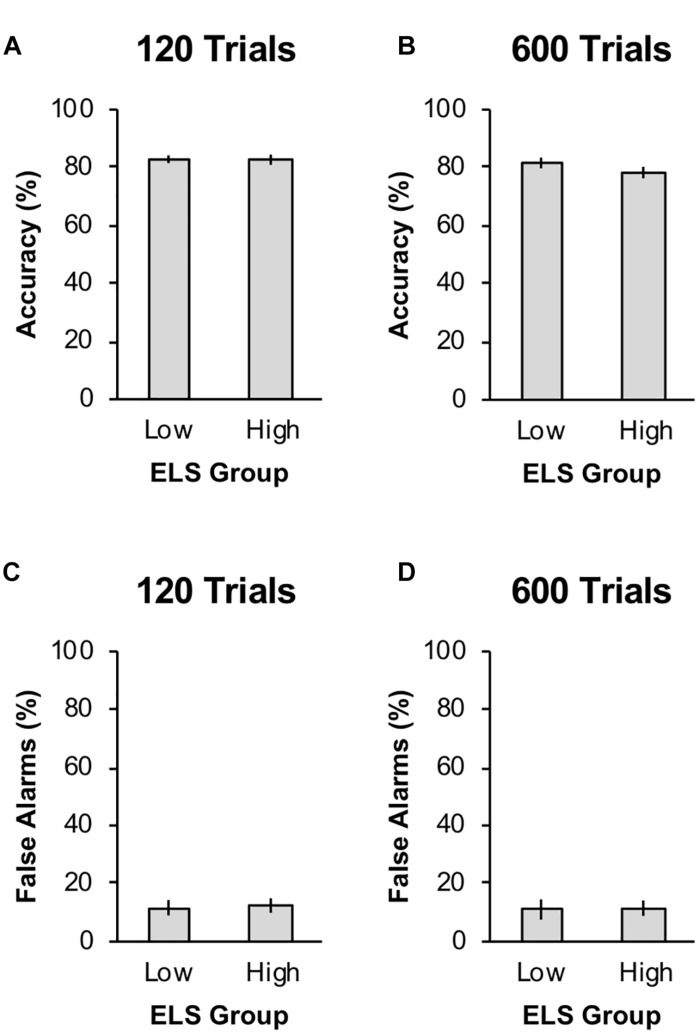
Acquisition behavior for the two early-life stress (ELS) groups in Experiment 1 by training condition. Panels **(A)** and **(B)** show % correct avoidance responses to the warning stimuli during the training phase for the short and long training conditions, respectively. Panels **(C)** and **(D)** show % false alarms to the safe stimulus during the training phase for the short and long training conditions, respectively. Error bars represent one standard error from the mean.

We first tested for effects of the level of training (120 training trials versus 600 training trials) and level of ELS (low-ELS versus high-ELS) on response accuracy during training, false alarm rate during training, and level of responding to the valued stimulus during the post-devaluation habit test. The data from the training phase are shown in Figure 2. During training, response accuracy to the two warning stimuli was 81.29% (*SD* = 12.10%), and the false alarm rate to the safe stimulus was 11.68% (*SD* = 19.14%). Training accuracy did not differ significantly across levels of training, *F*(1,185) = 2.57, *p* = 0.111, ${\text{η}}_{\text{p}}^{2}$ = 0.014, or levels of ELS, *F*(1,185) = 0.96, *p* = 0.330, ${\text{η}}_{\text{p}}^{2}$ = 0.005, and the interaction between training and ELS was not significant, *F*(1,185) = 0.78, *p* = 0.379, ${\text{η}}_{\text{p}}^{2}$ = 0.004. False alarm rate did not differ significantly across levels of training, *F*(1,185) = 0.07, *p* = 0.786, ${\text{η}}_{\text{p}}^{2}$ < 0.001, or levels of ELS, *F*(1,185) = 0.05, *p* = 0.832, ${\text{η}}_{\text{p}}^{2}$ < 0.001, and the interaction between training and ELS was not significant, *F*(1,185) < 0.01, *p* = 0.960, ${\text{η}}_{\text{p}}^{2}$ < 0.001.

During the post-devaluation habit test, 100% of participants responded to the valued stimulus (i.e., performed the valued response in the presence of the valued stimulus), with an average response rate of 90.69% (*SD* = 12.93%). Responding to the valued stimulus did not differ significantly across levels of training, *F*(1,185) = 2.77, *p* = 0.098, ${\text{η}}_{\text{p}}^{2}$ = 0.015, or levels of ELS, *F*(1,185) = 2.43, *p* = 0.121, ${\text{η}}_{\text{p}}^{2}$ = 0.013, and the interaction between training and ELS was not significant, *F*(1,185) = 1.13, *p* = 0.289, ${\text{η}}_{\text{p}}^{2}$ = 0.006.

**FIGURE 3:**
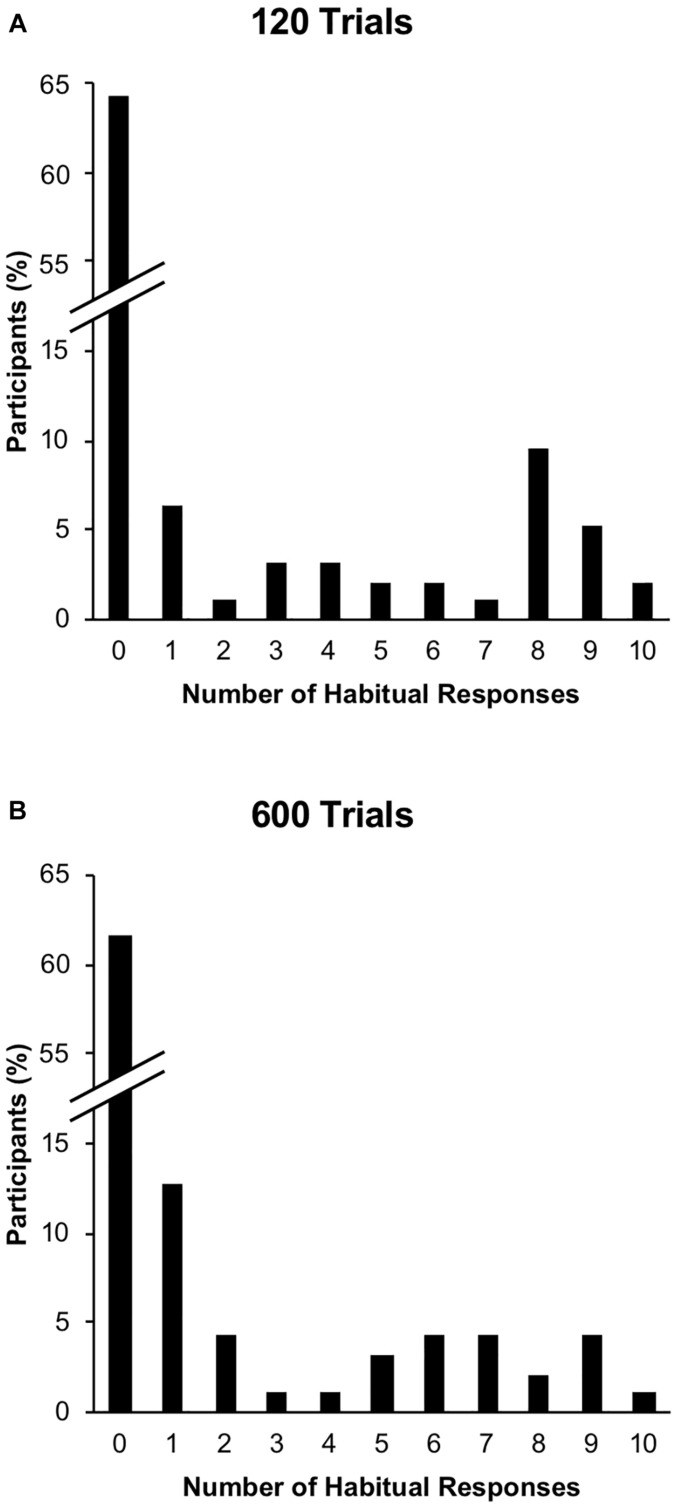
Distribution of responses to the devalued stimulus in Experiment 1. Panels **(A)** and **(B)** show % of participants making each of the possible number of responses to the devalued stimulus during the post-devaluation habit test for the short and long training conditions, respectively.

The distribution of responses to the devalued stimulus (i.e., performance of the devalued response in the presence of the devalued stimulus) during the post-devaluation habit test is shown in Figure 3. The average response rate to the devalued stimulus was 18.57% (*SD* = 30.99%). Participants occasionally made the valued response to the devalued stimulus (average response rate = 10.11%, *SD* = 17.41%); these responses were not treated as habitual as they did not reflect the stimulus–response association learned during training. We tested for the effects of ELS and length of training on habitual behavior by conducting a binary logistic regression analysis on responding to the devalued stimulus during the post-devaluation habit test. Participants’ responses were binned into zero responses to the devalued stimulus (no habitual behavior) or one or more responses to the devalued stimulus (habitual behavior). This analysis was conducted in order to test for the effect of ELS by using CTQ-SF as a continuous predictor variable while controlling for the effects of age, gender, and the other questionnaire variables which differed across the low-ELS and high-ELS groups. We included devalued side (i.e., whether the right or left earphone was removed during the post-devaluation habit test) as a predictor because there is evidence suggesting that individuals are more likely to engage in habitual behaviors when they are using their dominant hand (Neal et al., 2011). Although we did not measure participants’ handedness, it is reasonable to assume that a large majority of participants were right hand dominant and therefore might show greater habitual responding if assigned to the condition in which the right side was devalued. We also included interaction terms to test for moderation of the effect of ELS. The results of this analysis are shown in Table 3. Consistent with our hypothesis of greater habitual behavior in individuals with a history of stress during development, ELS was found to be a significantly positive predictor of habitual responding, *B* = 0.080, *p* = 0.020. The odds ratio for this predictor was 1.083, meaning that for every one point increase in CTQ-SF score, the expected odds of performing a habitual response are increased by 8.3%. Contrary to our hypothesis of greater habitual responding in participants who received more training trials, level of training was not a significant predictor of habitual responding, *B* = 0.015, *p* = 0.962, and devalued side was not a significant predictor of habitual responding, *B* = −0.073, *p* = 0.822. None of the other questionnaire variables (state anxiety, trait anxiety, depression, and perceived stress) were significant predictors, smallest *p* = 0.438, and the effects of age and gender were also not significant, smallest *p* = 0.367. We did not observe evidence for moderation of the effects of ELS as is shown by the lack of significance in the interaction predictors, smallest *p* = 0.092.

**TABLE 3:** Summary of binary logistic regression analysis predicting responding to the devalued stimulus during the post-devaluation habit test in Experiment 1.

**Variable**	***B***	***SE***	**Wald**	***p***	**Exp(*B*)**
**CTQ-SF**	**0.080**	**0.034**	**5.39**	**0.020**	**1.083**
Level of training (120 trials vs. 600 trials)	0.015	0.320	0.00	0.962	1.016
Devalued side (right vs. left)	–0.073	0.326	0.05	0.822	0.929
STAI state	–0.005	0.018	0.09	0.766	0.995
STAI trait	0.013	0.025	0.27	0.601	1.013
BDI-II	–0.017	0.028	0.37	0.542	0.983
PSS	0.032	0.041	0.60	0.438	1.032
CTQ-SF × length of training	–0.045	0.031	2.07	0.150	0.956
CTQ-SF × devalued side	–0.048	0.033	2.14	0.143	0.953
CTQ-SF × STAI state	0.001	0.002	0.09	0.762	1.001
CTQ-SF × STAI trait	–0.005	0.003	2.84	0.092	0.995
CTQ-SF × BDI-II	–0.001	0.002	0.12	0.730	0.999
CTQ-SF × PSS	0.006	0.004	1.84	0.175	1.006
Age	–0.085	0.094	0.81	0.367	0.919
Gender (male vs. female)	0.166	0.405	0.17	0.681	1.181

*Significant results (*p* < 0.05) shown in bold. For dichotomous predictors, the first term in parenthetical is the reference. CTQ-SF, Childhood Trauma Questionnaire – Short Form (Bernstein et al., 2003); STAI, State–Trait Anxiety Inventory (Spielberger, 1983); BDI-II, Beck Depression Inventory-II (Beck et al., 1996); PSS, Perceived Stress Scale (Cohen et al., 1983).*

## Experiment 2

In Experiment 1, we found support for the hypothesis that ELS is associated with enhanced avoidance habits. Given the number of predictors included in the model, however, there is a risk that the observed effect was the result of Type I error. Therefore, in Experiment 2, we sought to replicate the effect of ELS observed in Experiment 1, and we also added a condition in which participants performed the avoidance learning task under distraction to test the hypothesis that stimulus–response associations learned under distraction would result in greater habitual responding.

### Materials and Methods

#### Participants

As in Experiment 1, study participants were recruited from the undergraduate student population in the Psychology Department at the University of California, Los Angeles. Participants were compensated with credit toward partial fulfillment of course requirements. Study procedures were approved by the Institutional Review Board of the University of California, Los Angeles, and all participants provided written record of informed consent.

A total of 119 participants were recruited for the study. One participant failed to follow the instructions, one participant provided incomplete questionnaire data, and five participants were excluded for left-hand dominance (see the section “Design and Procedure” below), yielding a sample size of 112 (90 women, 22 men, *M*_age_ = 20.54 years, *SD*_age_ = 1.59 years, age range: 18–26 years).

#### Design and Procedure

Participants performed the avoidance learning task described above in Experiment 1. We manipulated the level of distraction within subjects during the training phase of the experiment by having participants perform a counting task during alternate blocks of 30 trials. During counting blocks, participants were randomly shown an image of a dog or a cat for 500 ms after each noise avoidance trial. They were instructed to count the cats and ignore the dogs. At the end of each counting block, participants were asked to report how many cats they had counted in the previous block. Before beginning the main experiment, participants completed practice trials on both the avoidance task and the counting task, and were allowed to repeat the practice trials if desired. To minimize task difficulty, we increased the response window for the noise avoidance task from 500 to 750 ms. Six stimulus images were used for the noise avoidance task, such that the same three stimuli were shown during all counting blocks and the other three stimuli were shown during non-counting blocks. Participants completed a total of 360 training trials (180 trials per level of distraction).

The devaluation procedure was the same as in Experiment 1, except that in Experiment 2 we instructed all participants to remove the right earphone for the habit test. Although the effect of devalued side and the interaction between devalued side and ELS in Experiment 1 were not significant, there was slightly greater habitual responding in participants instructed to remove the right earphone, and the effect of ELS on habitual responding was slightly stronger among participants instructed to remove the right earphone. Therefore, in order to maximize our ability to detect an effect in Experiment 2, we screened participants for right hand dominance and then tested for habitual behavior in the right hand by having participants remove the right earphone during the devaluation procedure. The post-devaluation habit test consisted of 60 trials, 30 containing stimuli that had been learned in the no-distraction condition and 30 containing stimuli that had been learned in the distraction condition. The 60 stimuli were presented in random order. Participants were not required to perform the counting task during the habit test. As in Experiment 1, the dependent variable of interest was whether the participant persisted in performing the response associated with avoiding aversive noise to the removed earphone.

Participants completed the experiment in a private testing room on a desktop computer. Stimulus presentation and response collection were implemented in E-Prime Standard (Version 2.0). Button press responses were made using the computer keyboard. Following completion of the computer task, participants completed the packet of questionnaires described above for Experiment 1. We additionally administered the Edinburgh Handedness Inventory (Oldfield, 1971) to screen for right hand dominance, using a cut point of 0. Seventeen participants who did not complete the handedness questionnaire were all included in the sample. The entire lab visit took approximately 1 h.

#### Data Analysis

Statistical analyses were performed using IBM SPSS Statistics (Version 25). Data from the acquisition phase (response accuracy to the two warning stimuli and false alarm rate to the safe stimulus) and level of responding to the valued stimulus during the habit test were analyzed using two (level of distraction: no-distraction, distraction) × two (level of ELS: low-ELS, high-ELS) mixed-model ANOVA with participants categorized as low-ELS or high-ELS based on a median split of the CTQ-SF scores. Responding to the devalued stimulus during the post-devaluation habit test was analyzed using a binary logistic regression generalized linear mixed model with level of distraction as a repeated measure. As in Experiment 1, participants’ responses were binned into zero responses to the devalued stimulus (no habitual behavior) or one or more responses to the devalued stimulus (habitual behavior). The following predictors were included in the generalized linear mixed model: CTQ-SF, level of distraction, STAI state anxiety, STAI trait anxiety, BDI-II, PSS, CTQ-SF × level of distraction, CTQ-SF × STAI state anxiety, CTQ-SF × STAI trait anxiety, CTQ-SF × BDI-II, CTQ-SF × PSS, age, and gender. Continuous predictors used to create interaction terms were mean-centered to reduce multicollinearity, and dichotomous predictor variables were dummy coded. A significance level of 0.05 was used for all statistical tests.

A supplemental data analysis in which the number of responses to the devalued stimulus was entered as the outcome variable is provided in Supplementary Table 2.

### Results

Sample characteristics are reported in Table 1 (prevalence of ELS by degree and type of stress reported) and Table 2 (scores on questionnaire variables for low-ELS and high-ELS participants). The mean CTQ-SF score was 34.83 (*SD* = 9.26) and the median CTQ-SF score was 32.00. The low-ELS group had a mean CTQ-SF score of 27.80 (*SD* = 1.72) and the high-ELS group had a mean CTQ-SF score of 41.15 (*SD* = 8.70). The high-ELS group differed significantly from the low-ELS group on measures of state anxiety, *t*(110) = 3.28, *p* = 0.001, *d* = 0.62; trait anxiety, *t*(110) = 3.71, *p* < 0.001, *d* = 0.70; depression, *t*(110) = 3.47, *p* = 0.001, *d* = 0.66; and perceived stress, *t*(110) = 3.01, *p* = 0.003, *d* = 0.57.

**FIGURE 4:**
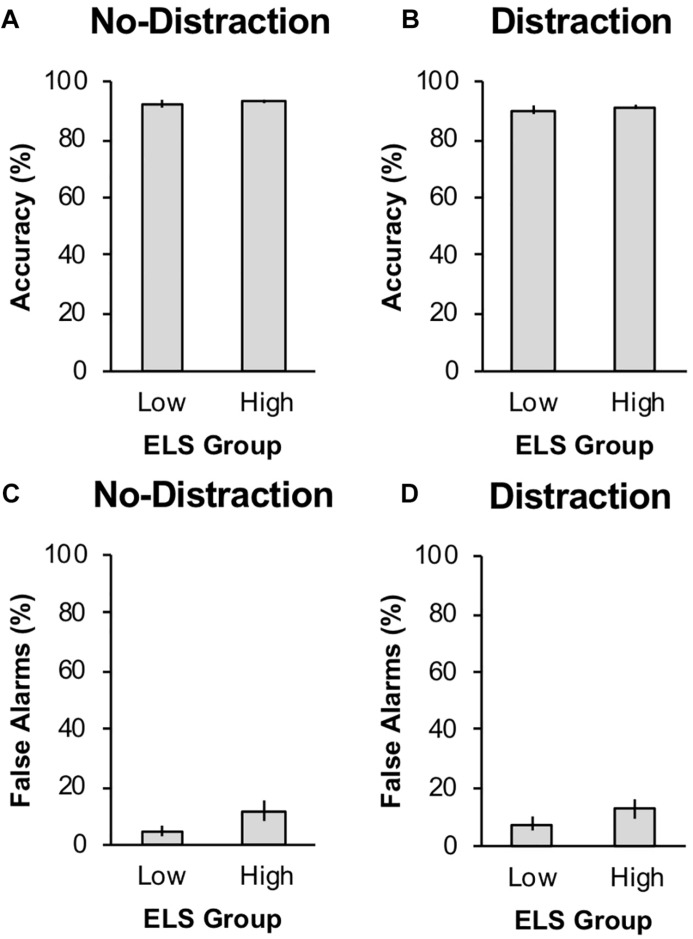
Acquisition behavior for the two early-life stress (ELS) groups in Experiment 2 by distraction condition. Panels **(A)** and **(B)** show % correct avoidance responses to the warning stimuli during the training phase for the no-distraction and distraction conditions, respectively. Panels **(C)** and **(D)** show % false alarms to the safe stimulus during the training phase for the no-distraction and distraction conditions, respectively. Error bars represent one standard error from the mean.

We first tested for effects of the level of distraction (no-distraction versus distraction) and level of ELS (low-ELS versus high-ELS) on response accuracy during training, false alarm rate during training, and level of responding to the valued stimuli during the post-devaluation habit test. The data from the training phase are shown in Figure 4. During training, response accuracy to the four warning stimuli was 91.74% (*SD* = 7.59%), and the false alarm rate to the two safe stimuli was 9.45% (*SD* = 19.13%). There was a significant effect of distraction on training accuracy, *F*(1,110) = 16.05, *p* < 0.001, ${\text{η}}_{\text{p}}^{2}$ = 0.127, such that accuracy was higher in single-task condition blocks (*M* = 92.72%, *SD* = 7.60%) than in dual-task condition blocks (*M* = 90.76%, *SD* = 8.41%). Training accuracy did not differ significantly across levels of ELS, *F*(1,110) = 0.41, *p* = 0.525, ${\text{η}}_{\text{p}}^{2}$ = 0.004, and the interaction between distraction and ELS was not significant, *F*(1,110) = 0.05, *p* = 0.830, ${\text{η}}_{\text{p}}^{2}$ < 0.001. False alarm rate did not differ significantly across levels of distraction, *F*(1,110) = 0.89, *p* = 0.349, ${\text{η}}_{\text{p}}^{2}$ = 0.008, or levels of ELS, *F*(1,110) = 2.76, *p* = 0.099, ${\text{η}}_{\text{p}}^{2}$ = 0.024, and the interaction between distraction and ELS was not significant, *F*(1,110) = 0.22, *p* = 0.637, ${\text{η}}_{\text{p}}^{2}$ = 0.002.

During the post-devaluation habit test, 100% of participants responded to the valued stimuli (i.e., performed the valued response in the presence of the valued stimuli), with an average response rate of 92.99% (*SD* = 10.75%). Responding to valued stimuli did not differ significantly across levels of distraction, *F*(1,110) = 0.07, *p* = 0.791, ${\text{η}}_{\text{p}}^{2}$ = 0.001, or levels of ELS, *F*(1,110) = 0.08, *p* = 0.773, ${\text{η}}_{\text{p}}^{2}$ = 0.001, and the interaction between distraction and ELS was not significant, *F*(1,110) = 1.56, *p* = 0.214, ${\text{η}}_{\text{p}}^{2}$ = 0.014.

**FIGURE 5:**
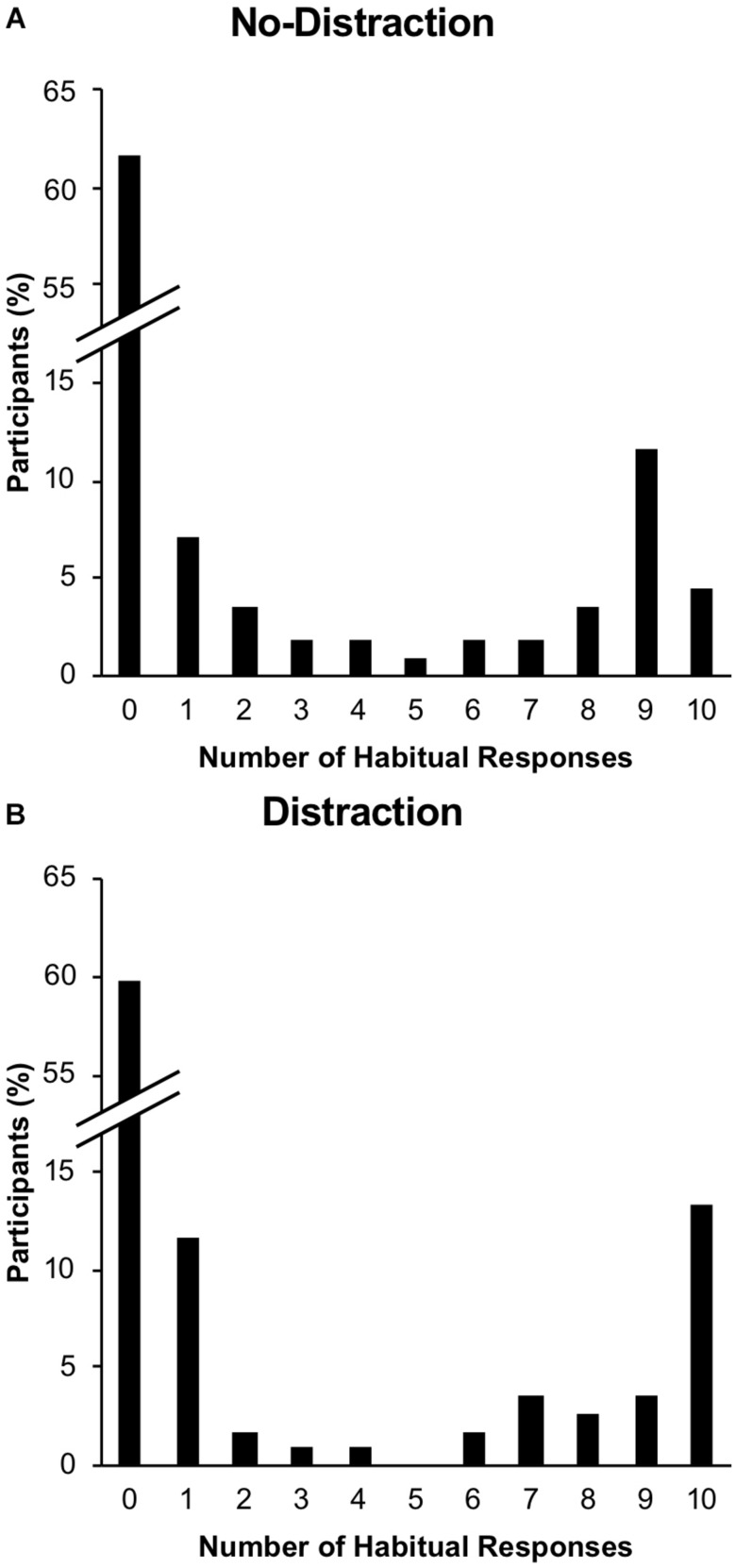
Distribution of responses to the devalued stimulus in Experiment 2. Panels **(A)** and **(B)** show % of participants making each of the possible number of responses to the devalued stimulus during the post-devaluation habit test for the no-distraction and distraction conditions, respectively.

The distribution of responses to the devalued stimuli (i.e., performance of the devalued response in the presence of the devalued stimuli) during the post-devaluation habit test is shown in Figure 5. The average response rate to the devalued stimuli was 23.84% (*SD* = 36.91%). Participants occasionally made the valued response to the devalued stimuli (average response rate = 9.87%, *SD* = 18.16%); these responses were not treated as habitual as they did not reflect the stimulus–response associations learned during training. We tested for the effects of ELS and distraction on habitual behavior by conducting a binary logistic regression generalized linear mixed model analysis on responding to the devalued stimulus during the post-devaluation habit test. As in Experiment 1, participants’ responses were binned into zero responses to the devalued stimulus (no habitual behavior) or one or more responses to the devalued stimulus (habitual behavior). The results of this analysis are shown in Table 4. Consistent with Experiment 1, ELS was found to be a significantly positive predictor of habitual responding, *B* = 0.064, *p* = 0.022. The odds ratio for this predictor was 1.066, meaning that for every one point increase in CTQ-SF score, the expected odds of performing a habitual response are increased by 6.6%. Contrary to our hypothesis of greater habitual responding to stimuli that were trained in the presence of distraction, level of distraction was not a significant predictor of habitual responding, *B* = 0.112, *p* = 0.723. Of the other questionnaire variables included as predictors (state anxiety, trait anxiety, depression, and perceived stress), two were significantly positive predictors of habitual responding: state anxiety, *B* = 0.047, *p* = 0.018 and perceived stress, *B* = 0.093, *p* = 0.025. For state anxiety, the odds ratio was 1.048, meaning that for every one point increase in the STAI state anxiety score, the expected odds of performing a habitual response are increased by 4.8%. For perceived stress, the odds ratio was 1.097, meaning that for every one point increase in the PSS score, the expected odds of performing a habitual response are increased by 9.7%. The other questionnaire variables were not significant predictors, smallest *p* = 0.209, and the effects of age and gender were also not significant, smallest *p* = 0.352. We did not observe evidence for moderation of the effects of ELS as is shown by the lack of significance in the interaction predictors, smallest *p* = 0.143.

**TABLE 4:** Summary of binary logistic regression generalized linear mixed model analysis predicting responding to the devalued stimulus during the post-devaluation habit test in Experiment 2.

**Variable**	***B***	***SE***	***t***	***p***	**Exp(*B*)**
**CTQ-SF**	**0.064**	**0.028**	**2.31**	**0.022**	**1.066**
Distraction (no distraction vs. distraction)	0.112	0.315	0.36	0.723	1.119
**STAI state**	**0.047**	**0.020**	**2.39**	**0.018**	**1.048**
STAI trait	−0.033	0.026	−1.26	0.209	0.968
BDI-II	−0.003	0.026	−0.13	0.899	0.997
**PSS**	**0.093**	**0.041**	**2.25**	**0.025**	**1.097**
CTQ-SF × distraction	−0.016	0.035	−0.45	0.655	0.984
CTQ-SF × STAI state	−0.003	0.002	−1.47	0.143	0.997
CTQ-SF × STAI trait	−0.004	0.003	−1.18	0.240	0.996
CTQ-SF × BDI-II	−0.001	0.003	−0.23	0.817	0.999
CTQ-SF × PSS	0.000	0.005	0.07	0.946	1.000
Age	−0.004	0.103	−0.04	0.966	0.996
Gender (male vs. female)	−0.384	0.412	−0.93	0.352	0.681

*Significant results (*p* < 0.05) shown in bold. For dichotomous predictors, the first term in parenthetical is the reference. CTQ-SF, Childhood Trauma Questionnaire – Short Form (Bernstein et al., 2003); STAI, State–Trait Anxiety Inventory (Spielberger, 1983); BDI-II, Beck Depression Inventory-II (Beck et al., 1996); PSS, Perceived Stress Scale (Cohen et al., 1983).*

## Discussion

In two experiments using an avoidance learning task, we observed evidence of enhanced avoidance habits in adults who reported a history of ELS. An important implication of this finding is that this behavioral tendency may contribute to the negative health outcomes commonly experienced by individuals with a history of ELS. Some of the negative health outcomes associated with self-reported developmental stress include severe obesity (Anda et al., 2006), heart disease (Dong et al., 2004), liver disease (Dong et al., 2003), and sexually transmitted disease (Hillis et al., 2000). Negative health outcomes are frequently tied to negative health behaviors, which may be performed habitually. Some of the negative health behaviors associated with self-reported developmental stress that contribute to the aforementioned negative health outcomes include smoking (Anda et al., 1999), alcohol abuse (Dube et al., 2002), and risky sexual behavior (Hillis et al., 2001). These negative health behaviors, along with the overeating that contributes to severe obesity and obesity-related health outcomes, can be conceptualized as avoidance behaviors, which over time can become avoidance habits. For example, individuals may initially engage in overeating, substance use, or risky sexual behavior in a goal-directed manner to avoid feelings of distress, but over time these behaviors may become more automatic and stimulus-bound. It should be noted, however, that such behaviors have an appetitive aspect to them as well; understanding the relationship between ELS and negative health outcomes may require a model in which behavior is driven by both appetitive and avoidant motivations, such as in Baumeister’s (1991) “escape from the self” theory of alcoholism.

One question raised by this pair of experiments is whether ELS is linked specifically to avoidance habits as opposed to avoidance behavior. Although we did not observe differences in avoidance behavior between the low-ELS and high-ELS participants during training, such differences may exist. Because the stimulus–response–outcome contingencies were demonstrated to participants explicitly at the beginning of the training phase rather than learned through experience, we may have had limited sensitivity to detect differences in the initial learning of the associations. This question could be tested in future research.

A possible biological basis for enhanced habitual behavior following ELS is that stress selectively compromises the neural structures that support goal-directed behavior, which could lead to a compensatory over-reliance on habitual responding. Goal-directed behavior relies on prefrontal cortex, dorsomedial striatum, and the hippocampus, which have been shown to atrophy following stress exposure (McEwen, 2000; Joëls et al., 2007; Dias-Ferreira et al., 2009; Soares et al., 2012). Habitual behavior, on the other hand, appears to rely on the dorsolateral striatum (Yin and Knowlton, 2004; Yin et al., 2004, 2006), which is less sensitive to stress and indeed has been shown in some cases to undergo stress-induced hypertrophy (Dias-Ferreira et al., 2009; Soares et al., 2012). The extent to which these morphological changes are reversible is not known. The presence of significant stress during a sensitive period of development may crystallize these dynamics, setting the stage for an overreliance on habitual responding in adulthood. Some evidence supporting this hypothesis includes the finding that male rats exposed to maternal separation during the first 2 weeks of life are more likely to use a stimulus–response navigation strategy in early adolescence (Grissom et al., 2012), and humans exposed to stress prenatally are more likely to use a stimulus–response navigation strategy in adulthood (Schwabe et al., 2012). Future research incorporating neuroimaging of habit learning in the ELS population should investigate this possibility. Recent neuroimaging studies that target the neuroendocrine basis of the stress-induced shift toward habitual behavior are helping to elucidate the mechanisms that underlie this shift (for review, see Wirz et al., 2018); it would be interesting to see how the effects of acute stress on habit compare to the effects of ELS on habit at the neural level.

An additional finding of the present study is that in Experiment 2 we also observed enhanced avoidance habits in individuals who reported higher levels of state anxiety and higher levels of perceived stress during the past month. This finding is consistent with previous literature on stress and habitual behavior (e.g., Schwabe and Wolf, 2009, 2010, 2012), but to our knowledge an effect of stress has not previously been demonstrated with avoidance habits. However, since this result was only present in Experiment 2 and not in Experiment 1, further research should be done to confirm the finding.

In addition to providing support for the hypothesis that ELS alters the tendency toward habitual responding, the results of the present study also demonstrate the utility of avoidance learning tasks in human habit research. Research on habits in humans has traditionally been carried out in appetitive situations with participants working for monetary rewards, points, or food (e.g., Tricomi et al., 2009), but tasks employing aversive stimuli have a long history of success in the non-human animal habit learning literature, particularly in maze navigation tasks where animals are motivated to escape a negative situation such as a water tank or open surface (e.g., Packard and McGaugh, 1992; McDonald and White, 1994). However, it should be noted that these maze navigation studies differ from the present study in that they do not use outcome devaluation to test habitual behavior. Aversive stimuli like the scream sound used in the present study are not difficult to incorporate into computer-based tasks and may provide greater motivation than appetitive stimuli.

Two hypotheses that we made in this pair of experiments were not borne out by the results. In Experiment 1, we predicted that a longer period of training would result in greater habitual responding, and in Experiment 2, we predicted that distraction during training would result in greater habitual responding. Neither of these manipulations affected the level of habit formation as measured by our post-devaluation habit test. It is possible that the manipulations we employed were not effective because the manipulations were not strong enough. Our manipulation of amount of training was a fivefold increase in the number of training trials, but participants in the long training condition still received only a single training session, and it is possible that to see an effect of training, multiple sessions would be required. A previous study conducted with appetitive stimuli that showed an effect of level of training on habitual responding implemented 12 training sessions over the course of 3 days (Tricomi et al., 2009); an avoidance learning study with a similar amount of training across multiple days may reveal a relationship between level of training and habitual responding. Similarly, the distraction task we used may have failed to provide enough of a challenge to produce the distraction-induced increase in habitual responding observed in previous studies (Foerde et al., 2006, 2007).

On the other hand, our failure to find an effect of length of training on habitual responding is consistent with a recent series of experiments conducted by de Wit et al. (2018), in which length of training was manipulated across a variety of tasks and in each case extended training failed to produce greater habitual responding. Notably, the noise avoidance procedure used in our experiments was very similar to the noise avoidance procedure used in one of the experiments conducted by de Wit et al. (2018); therefore, the null result of extended training in the present study serves as a replication of the null result of extended training reported in de Wit et al.’s (2018) noise avoidance experiment. The response rate to the devalued stimuli in our experiments was somewhat higher than the response rate to the devalued stimuli observed in the de Wit et al. (2018) noise avoidance experiment [approximately 20% in our experiments versus approximately 10% in the de Wit et al. (2018) experiment]. This difference may be due to the fact that responses in the de Wit et al. (2018) noise avoidance experiment were performed with a foot pedal whereas our participants performed responses with their index fingers on a computer keyboard.

An area of future research suggested by the present study is whether ELS affects habit learning, habit performance, or both. Previous research employing acute stress and challenges to executive control has indicated that these factors affect both the learning and performance of habits. For example, studies using the probabilistic classification task have shown that acute stress and distraction modulate which memory system is engaged during classification learning, biasing competition between the declarative memory system and the habit learning system in favor of habit learning (Foerde et al., 2006, 2007; Schwabe and Wolf, 2012). In contrast, studies that induce acute stress or executive challenge after learning but before a habit test demonstrate how these factors influence the performance of habits that have been learned previously. For example, Schwabe and Wolf (2010) showed that acute stress after learning decreases sensitivity to devaluation, and Lin et al. (2016) showed that completion of a task designed to deplete executive resources after learning an unhealthy habit increased performance of the unhealthy habit. Of course, as ELS cannot be induced between learning and testing, the paradigms that have been used to investigate performance effects of acute stress and executive challenge cannot be applied, and different paradigms possibly incorporating neuroimaging will be necessary to tease apart the effects of ELS on habit learning versus habit performance.

One limitation of the present study is that because we used a college sample, our ELS groups may be more high-functioning and resilient to stress than individuals with a history of ELS in the general population. Nevertheless, even this sample yielded evidence in support of our hypothesis that ELS affects avoidance habit formation. Future research with a more representative sample would, however, yield important information about the generalizability of our findings and typical effect sizes. A second limitation is that our sample was primarily composed of young adult females. Neither age nor sex were found to be significant predictors of habitual responding in this set of experiments; however, the age range of participants in our sample was relatively limited and the sample of male participants was relatively small. Future studies should investigate whether these variables are truly non-significant by testing a wider age range and sampling a larger number of males.

Our findings extend recent work demonstrating enhanced avoidance habits in individuals with obsessive-compulsive disorder (Gillan et al., 2014, 2015), identifying a second population with this behavioral pattern. Additional populations that may show similar patterns include individuals with post-traumatic stress disorder, binge eating disorder, and substance use disorders. Future research should investigate these possibilities. A deeper understanding of the role of avoidance habits in maladaptive behavior has the potential to inform interventions that may mitigate their negative effects on individuals’ lives.

## Data Availability

The datasets generated for this study are available on request to the corresponding author.

## Ethics Statement

This study was carried out in accordance with the recommendations of the Institutional Review Board of the University of California, Los Angeles with written informed consent from all subjects. All subjects gave written informed consent in accordance with the Declaration of Helsinki. The protocol was approved by the Institutional Review Board of the University of California, Los Angeles.

## Author Contributions

TP, MC, and BK contributed to the conception and design of the study. TP oversaw data acquisition, performed the statistical analysis, and wrote the first draft of the manuscript. All authors contributed to the manuscript revision, and read and approved the submitted version of the manuscript.

## Conflict of Interest Statement

The authors declare that the research was conducted in the absence of any commercial or financial relationships that could be construed as a potential conflict of interest.

## References

[B1] Adams C. D. (1982). Variations in the sensitivity of instrumental responding to reinforcer devaluation. *Q. J. Exp. Psychol. Section B* 34B 77–98. 10.1080/14640748208400878

[B2] Anda R. F. Croft J. B. Felitti V. J. Nordenberg D. Giles W. H. Williamson D. F. (1999). Adverse childhood experiences and smoking during adolescence and adulthood. *JAMA* 282 1652–1658. 10.1001/jama.282.17.1652 10553792

[B3] Anda R. F. Felitti V. J. Bremner J. D. Walker J. D. Whitfield C. Perry B. D. (2006). The enduring effects of abuse and related adverse experiences in childhood: a convergence of evidence from neurobiology and epidemiology. *Eur. Arch. Psychiatry Clin. Neurosci.* 256 174–186. 10.1007/s00406-005-0624-4 16311898 PMC3232061

[B4] Baumeister R. F. (1991). *Escaping the Self: Alcoholism, Spirituality, Masochism, and Other Flights from the Burden of Selfhood.* New York, NY: Basic Books.

[B5] Beck A. T. Steer R. A. Brown G. K. (1996). *Beck Depression Inventory*, 2 Edn San Antonio, TX: The Psychological Corporation.

[B6] Bernstein D. P. Stein J. A. Newcomb M. D. Walker E. Pogge D. Ahluvalia T. (2003). Development and validation of a brief screening version of the childhood trauma questionnaire. *Child Abuse Negl.* 27 169–190. 10.1016/S0145-2134(02)00541-0 12615092

[B7] Britton J. C. Lissek S. Grillon C. Norcross M. A. Pine D. S. (2011). Development of anxiety: the role of threat appraisal and fear learning. *Depress. Anxiety* 28 5–17. 10.1002/da.20733 20734364 PMC2995000

[B8] Chapman D. P. Whitfield C. L. Felitti V. J. Dube S. R. Edwards V. J. Anda R. F. (2004). Adverse childhood experiences and the risk of depressive disorders in adulthood. *J. Affect. Disord.* 82 217–225. 10.1016/j.jad.2003.12.013 15488250

[B9] Cohen S. Kamarck T. Mermelstein R. (1983). A global measure of perceived stress. *J. Health Soc. Behav.* 24 385–396. 10.2307/2136404 6668417

[B10] de Wit S. Kindt M. Knot S. L. Verhoeven A. A. C. Robbins T. W. Gasull-Camos J. (2018). Shifting the balance between goals and habits: five failures in experimental habit induction. *J. Exp. Psychol. Gen.* 147 1043–1065. 10.1037/xge0000402 29975092 PMC6033090

[B11] Dias-Ferreira E. Sousa J. C. Melo I. Morgado P. Mesquita A. R. Cerqueira J. J. (2009). Chronic stress causes frontostriatal reorganization and affects decision-making. *Science* 325 621–625. 10.1126/science.1171203 19644122

[B12] Dickinson A. (1985). Actions and habits: the development of behavioural autonomy. *Philos. Trans. Royal Soc. Lon., Series B Biol. Sci.* 308 67–78. 10.1098/rstb.1985.0010

[B13] Dong M. Dube S. R. Felitti V. J. Giles W. H. Anda R. F. (2003). Adverse childhood experiences and self-reported liver disease: new insights into the causal pathway. *Arch. Intern. Med.* 163 1949–1956. 10.1001/archinte.163.16.1949 12963569

[B14] Dong M. Giles W. H. Felitti V. J. Dube S. R. Williams J. E. Chapman D. P. (2004). Insights into causal pathways for ischemic heart disease: adverse childhood experiences study. *Circulation* 110 1761–1766. 10.1161/01.CIR.0000143074.54995.7F 15381652

[B15] Dube S. R. Anda R. F. Felitti V. J. Edwards V. J. Croft J. B. (2002). Adverse childhood experiences and personal alcohol abuse as an adult. *Addict. Behav.* 27 713–725. 10.1016/s0306-4603(01)00204-0 12201379

[B16] Foerde K. Knowlton B. J. Poldrack R. A. (2006). Modulation of competing memory systems by distraction. *Proc. Natl. Acad. Sci., U.S.A.* 103 11778–11783. 10.1073/pnas.0602659103 16868087 PMC1544246

[B17] Foerde K. Poldrack R. A. Knowlton B. J. (2007). Secondary-task effects on classification learning. *Mem. Cogn.* 35 864–874. 10.3758/bf03193461 17910172

[B18] Gillan C. M. Apergis-Schoute A. M. Morein-Zamir S. Urcelay G. P. Sule A. Fineberg N. A. (2015). Functional neuroimaging of avoidance habits in obsessive-compulsive disorder. *Am. J. Psychiatr.* 172 284–293. 10.1176/appi.ajp.2014.14040525 25526600 PMC4910868

[B19] Gillan C. M. Morein-Zamir S. Urcelay G. P. Sule A. Voon V. Apergis-Schoute A. M. (2014). Enhanced avoidance habits in obsessive-compulsive disorder. *Biol. Psychiatr.* 75 631–638. 10.1016/j.biopsych.2013.02.002 23510580 PMC3988923

[B20] Grissom E. M. Hawley W. R. Bromley-Dulfano S. S. Marino S. E. Stathopoulos N. G. Dohanich G. P. (2012). Learning strategy is influenced by trait anxiety and early rearing conditions in prepubertal male, but not prepubertal female rats. *Neurobiol. Learn. Mem.* 98 174–181. 10.1016/j.nlm.2012.06.001 22705447

[B21] Hillis S. D. Anda R. F. Felitti V. J. Marchbanks P. A. (2001). Adverse childhood experiences and sexual risk behaviors in women: a retrospective cohort study. *Fam. Plann. Perspect.* 33 206–211. 10.2307/2673783 11589541

[B22] Hillis S. D. Anda R. F. Felitti V. J. Nordenberg D. Marchbanks P. A. (2000). Adverse childhood experiences and sexually transmitted diseases in men and women: a retrospective study. *Pediatrics* 106 e11 1–6. 10.1542/peds.106.1.e11 10878180

[B23] Joëls M. Karst H. Krugers H. J. Lucassen P. J. (2007). Chronic stress: implications for neuronal morphology, function and neurogenesis. *Front. Neuroendocrinol.* 28 72–96. 10.1016/j.yfrne.2007.04.001 17544065

[B24] Kim J. J. Lee H. J. Han J.-S. Packard M. G. (2001). Amygdala is critical for stress-induced modulation of hippocampal long-term potentiation and learning. *J. Neurosci.* 21 5222–5228. 10.1523/jneurosci.21-14-05222.2001 11438597 PMC6762855

[B25] Knowlton B. J. Mangels J. A. Squire L. R. (1996). A neostriatal habit learning system in humans. *Science* 273 1399–1402. 10.1126/science.273.5280.1399 8703077

[B26] Knowlton B. J. Patterson T. K. (2018). “Habit formation and the striatum,” in *Behavioral Neuroscience of Learning and Memory. Current Topics in Behavioral Neurosciences* Vol. 37 eds Clark R. E. Martin S. J. (Cham: Springer), 275–295. 10.1007/7854_2016_451 27677776

[B27] Knowlton B. J. Squire L. R. Gluck M. A. (1994). Probabilistic classification learning in amnesia. *Learn. Mem.* 1 106–120. 10.1101/lm.1.2.106 10467589

[B28] Lau J. Y. F. Lissek S. Nelson E. E. Lee Y. Roberson-Nay R. Poeth K. (2008). Fear conditioning in adolescents with anxiety disorders: results from a novel experimental paradigm. *J. Am. Acad. Child Adolesc. Psychiatr.* 47 94–102. 10.1097/chi.0b01e31815a5f01 18174830 PMC2788509

[B29] Lin P.-Y. Wood W. Monterosso J. (2016). Healthy eating habits protect against temptations. *Appetite* 103 432–440. 10.1016/j.appet.2015.11.011 26585633

[B30] McDonald R. J. White N. M. (1994). Parallel information processing in the water maze: evidence for independent memory systems involving dorsal striatum and hippocampus. *Behav. Neural Biol.* 61 260–270. 10.1016/s0163-1047(05)80009-3 8067981

[B31] McEwen B. S. (2000). Effects of adverse experiences for brain structure and function. *Biol. Psychiatr.* 48 721–731. 10.1016/s0006-3223(00)00964-1 11063969

[B32] Neal D. T. Wood W. Wu M. Kurlander D. (2011). The pull of the past: when do habits persist despite conflict with motives? *Personal. Soc. Psychol. Bull.* 37 1428–1437. 10.1177/0146167211419863 21859902

[B33] Oldfield R. C. (1971). The assessment and analysis of handedness: the Edinburgh inventory. *Neuropsychologia* 9 97–113. 10.1016/0028-3932(71)90067-4 5146491

[B34] Packard M. G. McGaugh J. L. (1992). Double dissociation of fornix and caudate nucleus lesions on acquisition of two water maze tasks: further evidence for multiple memory systems. *Behav. Neurosci.* 106 439–446. 10.1037/0735-7044.106.3.439 1616610

[B35] Patterson T. K. (2018). *Effects of Early-Life Stress on Actions, Habits, and the Neural Systems Supporting Instrumental Behavior.* Los Angeles, CA: University of California, Los Angeles [dissertation].

[B36] Schwabe L. Bohbot V. D. Wolf O. T. (2012). Prenatal stress changes learning strategies in adulthood. *Hippocampus* 22 2136–2143. 10.1002/hipo.22034 22605683

[B37] Schwabe L. Dalm S. Schächinger H. Oitzl M. S. (2008). Chronic stress modulates the use of spatial and stimulus-response learning strategies in mice and man. *Neurobiol. Learn. Mem.* 90 495–503. 10.1016/j.nlm.2008.07.015 18707011

[B38] Schwabe L. Wolf O. T. (2009). Stress prompts habit behavior in humans. *J. Neurosci.* 29 7191–7198. 10.1523/JNEUROSCI.0979-09.2009 19494141 PMC6666491

[B39] Schwabe L. Wolf O. T. (2010). Socially evaluated cold pressor stress after instrumental learning favors habits over goal-directed action. *Psychoneuroendocrinology* 35 977–986. 10.1016/j.psyneuen.2009.12.010 20071096

[B40] Schwabe L. Wolf O. T. (2012). Stress modulates the engagement of multiple memory systems in classification learning. *J. Neurosci.* 32 11042–11049. 10.1523/jneurosci.1484-12.2012 22875937 PMC6621021

[B41] Seehagen S. Schneider S. Rudolph J. Ernst S. Zmyj N. (2015). Stress impairs cognitive flexibility in infants. *Proc. Natl. Acad. Sci. U.S.A.* 112 12882–12886. 10.1073/pnas.1508345112 26417100 PMC4611673

[B42] Soares J. M. Sampaio A. Ferreira L. M. Santos N. C. Marques F. Palha J. A. (2012). Stress-induced changes in human decision-making are reversible. *Trans. Psychiatr.* 2 e131 1–7. 10.1038/tp.2012.59 22760555 PMC3410630

[B43] Spielberger C. D. (1983). *Manual for the State-Trait Anxiety Inventory (Form Y).* Menlo Park, CA: Mind Garden.

[B44] Tricomi E. Balleine B. W. O’Doherty J. P. (2009). A specific role for posterior dorsolateral striatum in human habit learning. *Eur. J. Neurosci.* 29 2225–2232. 10.1111/j.1460-9568.2009.06796.x 19490086 PMC2758609

[B45] Wirz L. Bogdanov M. Schwabe L. (2018). Habits under stress: mechanistic insights across different types of learning. *Curr. Opin. Behav. Sci.* 20 9–16. 10.1016/j.cobeha.2017.08.009

[B46] Yin H. H. Knowlton B. J. (2004). Contributions of striatal subregions to place and response learning. *Learn. Mem.* 11 459–463. 10.1101/lm.81004 15286184 PMC498333

[B47] Yin H. H. Knowlton B. J. Balleine B. W. (2004). Lesions of dorsolateral striatum preserve outcome expectancy but disrupt habit formation in instrumental learning. *Eur. J. Neurosci.* 19 181–189. 10.1111/j.1460-9568.2004.03095.x 14750976

[B48] Yin H. H. Knowlton B. J. Balleine B. W. (2006). Inactivation of dorsolateral striatum enhances sensitivity to changes in the action–outcome contingency in instrumental conditioning. *Behav. Brain Res.* 166 189–196. 10.1016/j.bbr.2005.07.012 16153716

