# Supplementary material for: Enhanced Avoidance Habits in Relation to History of Early-Life Stress
Source: Front Psychol. 2019 Aug 13;10:1876. doi: 10.3389/fpsyg.2019.01876 (PMC6700232; doi:10.3389/fpsyg.2019.01876)
Supplement: Supplementary file 1 [file Data_Sheet_1.PDF]

Supplementary Material

Supplementary Table 1. Summary of regression analysis predicting number of responses to the devalued stimulus during the post-devaluation habit test in Experiment 1.

Variable	*B*	*SE*	*t*	*p*
CTQ-SF	0.081	0.048	1.70	.090
Level of training (120 trials vs. 600 trials)	-0.410	0.465	-0.88	.378
Devalued side (right vs. left)	-0.719	0.472	-1.52	.130
STAI state	-0.034	0.027	-1.26	.209
STAI trait	0.061	0.036	1.69	.093
BDI-II	-0.005	0.041	-0.13	.898
PSS	0.020	0.059	0.33	.741
CTQ-SF × length of training	-0.079	0.043	-1.82	.070
CTQ-SF × devalued side	-0.057	0.045	-1.29	.200
CTQ-SF × STAI state	0.000	0.002	0.21	.835
CTQ-SF × STAI trait	-0.004	0.004	-1.07	.287
CTQ-SF × BDI-II	0.000	0.003	-0.05	.964
CTQ-SF × PSS	0.004	0.006	0.72	.475
Age	-0.070	0.133	-0.53	.598
Gender (male vs. female)	-0.167	0.578	-0.29	.773

Note. For dichotomous predictors, the first term in parenthetical is the reference. CTQ-SF = Childhood Trauma Questionnaire – Short Form (Bernstein et al., 2003); STAI = State-Trait Anxiety Inventory (Spielberger, 1983); BDI-II = Beck Depression Inventory-II (Beck et al., 1996); PSS = Perceived Stress Scale (Cohen et al., 1983).

Supplementary Table 2. Summary of generalized linear mixed model analysis predicting number of responses to the devalued stimulus during the post-devaluation habit test in Experiment 2.

Variable	*B*	*SE*	*t*	*p*
CTQ-SF	0.075	0.039	1.952	.052
Distraction (no distraction vs. distraction)	0.121	0.460	0.262	.793
STAI state	0.084	0.028	2.976	.003
STAI trait	-0.027	0.039	-0.679	.498
BDI-II	-0.053	0.040	-1.334	.184
PSS	0.134	0.060	2.231	.027
CTQ-SF × distraction	-0.023	0.050	-0.453	.651
CTQ-SF × STAI state	-0.006	0.003	-1.971	.050
CTQ-SF × STAI trait	0.000	0.005	-0.097	.923
CTQ-SF × BDI-II	0.001	0.005	0.277	.782
CTQ-SF × PSS	0.004	0.007	0.546	.586
Age	-0.081	0.154	-0.522	.602
Gender (male vs. female)	-1.151	0.608	-1.893	.060

Note. For dichotomous predictors, the first term in parenthetical is the reference. CTQ-SF = Childhood Trauma Questionnaire – Short Form (Bernstein et al., 2003); STAI = State-Trait Anxiety Inventory (Spielberger, 1983); BDI-II = Beck Depression Inventory-II (Beck et al., 1996); PSS = Perceived Stress Scale (Cohen et al., 1983).
